# Sapiens Parque: the innovation park of the technology pole of Greater Florianópolis for the promotion of sectors that propel the future

**DOI:** 10.1186/1753-6561-8-S4-O49

**Published:** 2014-10-01

**Authors:** Carlos Alberto Schneider

**Affiliations:** 1 Fundação CERTI, Florianópolis - SC, 88040-970, Brazil

## Technology pole of Greater Florianópolis

Competitive companies are one of the bases of a nation's economic development. Among the proven success strategies in this field are Scientific and Technology Parks, whose mission is to promote intelligence that expands corporate competitiveness.

In this context, Florianópolis stands out in Brazil for its development based on the promotion of innovation. This strategy was materialized by a policy called TECNÓPOLIS that induced the creation of companies by establishing facilities that support innovation like the CELTA incubator, ParqTec Alfa and the innovative Sapiens Parque.

## Sapiens and its concept

Sapiens Parque is the fruit of research and development. It is characterized as an Innovation Park, which at the time of it launching in April 2003 was a globally unprecedented concept that is described below:

An Innovation Park is a ***Cluster of Innovation Clusters *- **an **environment **fortified with **infrastructure and systems**to attract and develop **talents and enterprises **that are capable of generating **ideas and knowledge **and transform them into new **products and services**for society, promoting the region's **sustainable socio-economic development**.

The key instrument for Sapien's planning, implementation and operation is its Conceptual Model, which defines and integrates all the critical elements needed for guaranteeing the proper implementation of the development, as illustrated: (Fiates, Fiates/2011):

**1. "Sapiens Assets" **- these are the "pillars" of Sapiens, upon which the other subsystems are structured. The modules include: **Scientia, Artis, Naturallium and Gens**.

**2. "Sapiens Clusters" - **The Sapiens clusters establish its identity and "thematic territory" and operate in sectors that are strategic to the region, including **Technology, Tourism, Services and the Public Sector**.

**3. "Sapiens Structure" **- includes the physical, human and financial elements that support the operation of the clusters such as: the **Park Infrastructure**, **Regional Infrastructure, People and Capital**.

**4. "Sapiens Actors " - **these are the "Actors" with whom the Park should interact and for whom the results of the development are generated. They include: **government, companies, research and education institutions and society**.

1. Formation of Innovation ClustersConsidering local and regional factors and based on state and national studies, four priority areas of operation were defined for Sapiens: Clean Energy & Technologies, Health & Biotechnology, Creative Economy and ICT & Mechatronics. These are the embryos of the so-called Innovation Clusters at Sapiens.

## Final considerations

Sapiens Parque is a large and long term development conceived to establish a new growth cycle in Greater Florianopolis' Innovation Pole. It is organized from a legal and business perspective to permit agile and consistent public and private investment, facilitate the transformation of knowledge and ideas into successful results in the market and consequently promote economic development throughout the region.
